# State- and Circuit-Dependent Opponent Processing of Fear

**DOI:** 10.1523/JNEUROSCI.0857-24.2024

**Published:** 2024-07-26

**Authors:** Joanna Oi-Yue Yau, Amy Li, Lauren Abdallah, Leszek Lisowksi, Gavan P. McNally

**Affiliations:** ^1^School of Psychology, University of New South Wales, Sydney, New South Wales 2052, Australia; ^2^Translational Vectorology Research Unit, Children’s Medical Research Institute, Faculty of Medicine and Health, The University of Sydney, Westmead, New South Wales 2145, Australia; ^3^Australian Genome Therapeutics Centre, Children’s Medical Research Institute and Sydney Children’s Hospitals Network, Westmead, New South Wales 2145, Australia; ^4^Laboratory of Molecular Oncology and Innovative Therapies, Military Institute of Medicine - National Research Institute, Szaserów 128 Street, 04-141 Warszawa 44, Poland

**Keywords:** accumbens, amygdala, fear conditioning, photometry, relief, reward

## Abstract

The presence of valence coding neurons in the basolateral amygdala (BLA) that form distinct projections to other brain regions implies functional opposition between aversion and reward during learning. However, evidence for opponent interactions in fear learning is sparse and may only be apparent under certain conditions. Here we test this possibility by studying the roles of the BLA→central amygdala (CeA) and BLA→nucleus accumbens (Acb) pathways in fear learning in male rats. First, we assessed the organization of these pathways in the rat brain. BLA→CeA and BLA→Acb pathways were largely segregated in the BLA but shared overlapping molecular profiles. Then we assessed activity of the BLA→CeA and BLA→Acb pathways during two different forms of fear learning—fear learning in a neutral context and fear learning in a reward context. BLA→CeA neurons were robustly recruited by footshock regardless of where fear learning occurred, whereas recruitment of BLA→Acb neurons was state-dependent because footshock only recruited this pathway in a reward context. Finally, we assessed the causal roles of activity in these pathways in fear learning. Photoinhibition of the BLA→CeA pathway during the footshock US impaired fear learning, regardless of where fear learning occurred. In contrast, photoinhibition of the BLA→Acb pathway augmented fear learning, but only in the reward context. Taken together, our findings show circuit- and state-dependent opponent processing of fear. Footshock activity in the BLA→Acb pathway limits how much fear is learned.

## Significance Statement

Here we identify a fear opponent process in the brain. We show that an aversive event can recruit distinct populations of neurons in the rat basolateral amygdala. One population projects to the central amygdala to promote fear learning. A second population projects to the nucleus accumbens (Acb) to oppose fear learning. These Acb-projecting neurons limit how much fear is learned and are candidates for therapeutic targeting to minimize the amount of fear learned after a traumatic experience.

## Introduction

The basolateral amygdala (BLA) enables learning about emotional events by encoding their valence, assigning that valence to their predictors, and selecting appropriate behavioral responses (e.g., approach vs avoidance; Janak and Tye, 2015; Tye, 2018). These roles are supported by a complex mosaic of BLA neurons. In mice, BLA principal glutamatergic neurons can be distinguished on the basis of their long-range projections. BLA neurons projecting to the central amygdala (CeA) respond preferentially to aversive events (e.g., quinine, footshock) and their predictors, whereas BLA neurons projecting to the nucleus accumbens (Acb) respond preferentially to rewarding events (e.g., sucrose) and their predictors (Beyeler et al., 2016). BLA principal neuron molecular heterogeneity overlaps with these circuit features and may contribute to valence and learning. For example, BLA principal neurons expressing *Ppp1r1b* have been linked to positive valence, whereas neurons expressing *Rspo2* have been linked to negative valence (Kim et al., 2016). There are important nuances within this general BLA mosaic. For example, a subpopulation of *Rspo2* BLA neurons expressing *Fezf2* encode positive versus negative valence based on their long-range projections to the Acb and olfactory tubercle (Zhang et al., 2021).

The presence and organization of BLA valence coding circuits are suggestive of opponent processing of learning, where an opponent (e.g., reward or relief) process is recruited during learning to limit how much fear is learned (Konorski, 1967; Solomon and Corbit, 1974; Schull, 1979). This possibility is supported by findings that positive and negative valence BLA neurons can mutually inhibit each other, most likely via parvalbumin interneurons (Kim et al., 2016; Piantadosi et al., 2024). However, despite compelling evidence for opponent processes at the behavioral level, as shown via phenomena such as superconditioning and counterconditioning (Dickinson and Dearing, 1979; Nasser and McNally, 2012, 2013), evidence for opposition between BLA circuits during fear learning is sparse. We do know that the BLA→CeA pathway is important for fear learning because optogenetic inhibition of BLA→CeA neurons impairs fear learning (Namburi et al., 2015). However, optogenetic inhibition of the reward coding BLA→Acb pathway, which an opponent process model predicts should augment fear learning, had no effect on fear learning (Namburi et al., 2015).

So, if there is functional opposition between BLA aversion and reward coding circuits, this opposition may only be apparent under certain conditions. Here, we test these ideas by studying the roles of the BLA→CeA and BLA→Acb pathways in fear learning in rats. First, we assessed the anatomical and molecular organization of these pathways in the rat brain. Then we assessed activity of these pathways during two different forms of fear learning—fear learning in a neutral context and fear learning in a reward context. Finally, we assessed the causal roles of activity in these pathways in fear learning in a neutral context and a reward context. Our findings show, for the first time, circuit- and state-dependent opponent processing of fear. Footshock-driven activity in the BLA→Acb pathway can functionally oppose the BLA→CeA pathway to limit how much fear is learned.

## Materials and Methods

### Subjects

Subjects were experimentally naive male Long–Evans rats (280–500 g) obtained from the Animal Resource Centre and from The University of New South Wales. Female rats were not available, and we consider this limitation in Discussion. Rats were housed in groups of 2–4 in a climate-controlled colony room. Lights were on at 7:00A.M. and off at 7:00P.M., with all experiments conducted in the light cycle. Animals were allowed *ad libitum* access to chow and water unless otherwise stated.

### Behavioral apparatus

Behavioral training was conducted in identical Med Associates chambers. The chambers were 24 cm (length) × 30 cm (width) × 21 cm (height) and enclosed in a ventilated, sound-attenuating cabinets measuring 59.5 cm (length) × 59 cm (width) × 48 cm (height). The left sidewall was fitted with a magazine dish where grain pellets (Bio-Serv) were sometimes delivered when a retractable lever located 4 cm to the right of the magazine was pressed. A 3 W house light was mounted on top of the right sidewall next to a speaker used to deliver auditory conditioned stimuli (CSs). A metal grid was fitted to the floor of the chamber to deliver a scrambled footshock unconditioned stimulus (US). For optogenetics experiments, an LED with an integrated rotary joint (Doric Instruments) was suspended above the chamber and controlled by an LED driver connected to Med Associates.

### Retrograde tracers and viral vectors

Cholera toxin subunit B (CTb; recombinant) Alexa Fluor 488 (catalog #C34775, Thermo Fisher Scientific), and CTb Alexa Fluor 555 (catalog #CC34776, Thermo Fisher Scientific) were used for anatomical and molecular investigation of BLA output pathways. AAVs driven from a CamKIIα promoter were used to globally target BLA principal neurons, whereas retrograde AAVs were used to target specific BLA pathways. We used AAV5-CaMKIIα-eNpHR3.0-eYFP (6 × 10^12^) and AAV5-CaMKIIα-eYFP (4 × 10^12^; both from University of North Carolina Vector Core) to target BLA neurons. We packaged pAAV-hSyn-eNpHR 3.0-EYFP [a gift from Karl Deisseroth (Addgene plasmid 26972; http://n2t.net/addgene:26972; RRID:Addgene_26972)] and pAAV-hSyn-EGFP [a gift from Bryan Roth (Addgene plasmid 50465; http://n2t.net/addgene:50465; RRID:Addgene_50465)] into a retrograde AAV helper vector [a gift from Alla Karpova and David Schaffer (Addgene plasmid 81070; http://n2t.net/addgene:81070; RRID:Addgene_81070); Tervo et al., 2016], at the Vector Genome Facility (Westmead Children's Hospital) to obtain AAV2_retro_-hSyn-eNpHR3.0-eYFP (2.96 × 10^13^) and AAV_retro_-hSyn-eGFP (1 × 10^13^). We used AAVretrograde-gCaMP7f [1.8 × 10^13^; pGP-AAV-syn-jGCaMP7f-WPRE was a gift from Douglas Kim and GENIE Project (Addgene viral prep 104488-AAVrg; http://n2t.net/addgene:104488; RRID:Addgene_104488)] and AAVretrograde-jRGECO1a [7 × 10^12^; pAAV.Syn.NES-jRGECO1a.WPRE.SV40 was a gift from Douglas Kim and GENIE Project (Addgene viral prep 100854-AAVrg; http://n2t.net/addgene:100854; RRID:Addgene_100854); Dana et al., 2016].

### Surgery

Rats were anesthetized with isoflurane (5% induction; 2% maintenance) mixed with oxygen and placed in a stereotaxic frame (David Kopf Instruments). Prior to incision, rats received the analgesic carprofen (Rimadyl, Zoetis; 5 mg/kg) via a subcutaneous injection and 0.5% bupivacaine under the incision site. Following an incision to expose the skull, a hand drill was used to make a craniotomy above each injection and cannula implantation site. A 23 gauge, cone-tipped 5 ml stainless-steel injector (SGE Analytical Science) holding tracer or AAV was lowered into each injection site and infused at a rate of 100 nl/min (UltraMicro Pump III with SYS-Micro4 Controller, World Precision Instruments). For CeA, these coordinates were −2.25 (AP), ±4.25 (ML), and −7.9 (DV). For Acb, these coordinates were +1.2 to 1.3 (AP), ±1.5 or ±1.7 at 6° (ML), and 7.5 (DV). For BLA, these coordinates were −3.24 (AP), ±5.15 (ML), and −8.1 (DV). All coordinates are in millimeter from the bregma. The injector was left in place for additional 7 min for diffusion. For photometry and optogenetics experiments, a 400 µm fiber-optic cannula was implanted unilaterally (photometry) or bilaterally (optogenetics) above the BLA (AP, −3.24; ML, ±5.1; DV, −7.9, in millimeter from the bregma) and secured in place by dental cement (Vertex Dental) anchored to jeweller's screws attached to the skull. The incision was sutured, and antibiotic (Duplocillin, Intervet; 40,000–60,000 IU/kg) was given intraperitoneally. Rats were monitored until the end of the experiment.

We used CTb Alexa Fluor 488 or Alexa Fluor 555 conjugate to label CeA- and Acb-projecting BLA cells in the same rat. One variant was injected into the CeA, and the other was injected into the Acb of the same hemisphere. Each injection was 200 nl in volume. The tracer color variant and the injected hemisphere were counterbalanced across rats.

We used unilateral injection of the AAV containing CaMKIIα-gCaMP7f (fiber photometry) or bilateral injection of the AAV-CaMKIIα-eNpHR3.0-eYFP or the control AAV-CaMKIIα-eYFP (optogenetics) to target BLA glutamatergic neurons nonselectively. Each injection was 750 nl.

We used retrograde AAVs containing the green- (gCaMP7f; Dana et al., 2019) or red-shifted (jRGECO1a; Dana et al., 2016) calcium sensor to target BLA output pathways for simultaneous recording. Each rat received three craniotomies in the same hemisphere—one above the CeA, one above the Acb, and one above the BLA. One sensor was injected into the CeA at a volume of 300 nl, and the other was injected into the AcbSh at a volume of 500 nl. The sensor used at each projection site and the hemisphere injected were counterbalanced.

We used retrograde AAVs containing either eNpHR3.0 or the control eGFP to photoinhibit the BLA→CeA and BLA→Acb pathways. Rats received bilateral AAV injections into the CeA (100 nl injection) or Acb (750 nl injection).

### Single-molecule FISH

Brains were extracted 7 d after CTb injections, snap-frozen with liquid nitrogen, and stored at −80°C. Brains were sliced coronally at 10 µm, and the BLA was collected onto superfrosted microscope slides. Accumbens and amygdala regions were also collected on separate slides to confirm CTb injection sites. We used an RNAscope Fluorescent HiPlex Kit (Advanced Cell Diagnostics) with *Rspo2* (ADV1190711T6), *Ppp1r1b* (ADV1048941T9), and *Fezf2* (ADV1190731T12) probes custom designed for rat. BLA samples were treated with paraformaldehyde, dehydrated with ethanol and treated with Protease IV for 20 min. Slides were incubated with target probes, and each signal was sequentially amplified using HiPlex Amp 1–3. Dylight650 was applied to first label *Rspo2* and counterstained with DAPI. Slides were coverslipped, and DAPI, CTb 488, CTb555, and Dylight650 channels were imaged using an Axioscan 7 slide scanner (Carl Zeiss). The next day, coverslips were removed, and Dylight650 was cleaved and reattached to label *Ppp1r1b*, and the four channels were reimaged. This process was repeated once more to label and image *Fezf2*-expressing cells. Images were then collated using the ZEN software (Carl Zeiss), and labeled cells in the BLA were counted using CellProfiler (Stirling et al., 2021).

### Behavioral procedures

#### Fiber photometry

Rats were food restricted to ∼85% of their body weight 2 d prior to behavioral training, and they were maintained on this feeding regime until the end of the experiment. Rats received training in two separate contexts each day. In one context (reward context), a lever was available to earn a food pellet reward delivered into a magazine. In the second context (neutral context), a slate was slotted into the wall to block access to the magazine and to the lever slot. These contexts were further distinguished by olfactory (peppermint, rosewater), auditory (fan on or off), visual (houselight on or off), as well as spatial (different box with same dimensions in a different location) cues which were counterbalanced across rats.

On Days 1–5, rats received daily lever press training in the reward context and were placed in neutral context for the same duration. Context order was randomized each day. On Days 1–2, rats received magazine training in the reward context. In these sessions, every lever press was rewarded by grain pellet delivery, with additional “free” grain pellets delivered on a FI300 schedule. These free grain pellets facilitated learning of the lever press task. They were present only during Sessions 1 and 2. For Sessions 1 and 2 only, sessions were terminated when the rat reached 100 lever presses or after 60 min had elapsed. On Day 3, rats lever pressed for pellets on a VI30 schedule for 1 h. In all remaining sessions in the reward context, rats lever pressed for pellets on a VI120 schedule in a 1 h session. On Day 6, rats were pre-exposed to one auditory CS in the neutral context and a different auditory CS in the appetitive context. Auditory CSs were either a 60 s 10 Hz clicker or 60 s tone counterbalanced across context and rats. In each context, the 60 s CS was presented four times on an intertrial interval (ITI) of 600–900 s during a 1 h session. On Day 7, rats were tethered to patch cables and then subjected to fear conditioning in both contexts. In each context, the CS was presented four times, and each presentation coterminated with the 0.5 mA, 0.5 s footshock US during the 1 h session.

Recordings were made using the fiber photometry system from Doric Lenses and Tucker-Davis Technologies (RZ5P, Synapse). For CaMkIIα-gCaMP recordings, 465 and 405 nm wavelength light was emitted from LEDs controlled by programmable LED drivers. For dual-color, simultaneous recordings of BLA output pathways, an additional 560 nm wavelength light was emitted. Excitation lights were channeled through patch cables (0.39NA, Ø400 mm core multimode prebleached patch cables), and light intensity at the tip of the cable was maintained at 10–13 µW across sessions. Ca^2+^-dependent and isosbestic fluorescence were amplified and measured by Doric Fluorescence Detectors. The Synapse software controlled and modulated excitation lights (465 nm, 209 Hz; 405 nm, 331 Hz; 560 nm, 537 Hz) as well as demodulated and low-pass filtered (3 Hz) transduced fluorescence signals in real time via the RZ5P. RZ5P/Synapse also received Med-PC signals to record behavioral events in real time.

#### Photoinhibition during fear learning in reward context

In separate experiments, we assessed the roles of shock US-evoked activity in BLA, BLA→CeA pathway, and BLA→Acb pathways in fear learning in the reward context. Rats were first restricted to ∼85% of their body weight 2 d prior to behavioral training and were maintained on this schedule until the end of behavioral experimentation. Rats received one training session per day. On Days 1–2, rats received magazine training as described above. On Day 3, rats lever pressed for pellets on a VI30 schedule in a 2 h session. From Day 4 onward, rats were maintained on a VI120 schedule during daily 2 h sessions. From Day 7 onward, rats were tethered to patch cables. Rats received pre-exposure to the auditory CS (85 dB 10 Hz clicker) on Days 9–10 while lever pressing. In each 2 h session, rats received four 60 s presentations of the clicker CS with an ITI between 1,200 and 1,800 s. Rats were fear conditioned to the CS on Days 11–13. Prior to each 2 h session, rats were tethered to patch cables connected to a 625 nm LED controlled by an LED driver programmed via Med Associates. Rats received four presentations of the CS on a randomized ITI ranging between 1,200 and 1,800 s. Each CS coterminated with a 0.5 mA, 0.5 s footshock US. Photoinhibition occurred at the time of the US onset and continued for 4.5 s after the US offset. Rats were tested for their fear on Days 14–17. In each 70 min test session, rats were presented with four nonreinforced presentations of the CS on an ITI of 900 s.

#### Photoinhibition during fear learning in neutral context

In a separate experiment in a new cohort of animals, we assessed the role of the BLA→Acb pathway in fear learning in the neutral context; on Day 1, animals were pre-exposed to the shock context for 10 min while tethered to dummy patch cables. No stimuli were presented. On Day 2, rats underwent a 20 min fear conditioning involving three presentations of the 60 s auditory CS (85 dB 10 Hz clicker) at 120 s ITI that either coterminated with a 0.5 s 0.35 mA or 0.7 mA footshock US. We used three CS–US pairings because these were sufficient to reach asymptotic levels of freezing. Photoinhibition occurred at the time of the US onset and continued for 4.5 s after the US offset, and for 4.5 s thereafter, animals received constant optical stimulation of 625 nm wavelength light. On Days 3 and 4, rats were tethered to dummy patch cables and tested for their fear to the CS in 20 min sessions. They received six nonreinforced presentations of the 60 s CS at an ITI of 120 s.

### Histology

For retrograde tracing studies, rats were perfused 10 d after surgery, and brains were extracted and sliced coronally at 40 µm using a cryostat (Leica Microsystems). The BLA was collected onto slides, dried, and coverslipped. Four BLA sections were examined from each animal: one anterior (−2.40 mm to −2.64 mm from the bregma), one middle (−2.92 to −3.12 mm from the bregma), and two posterior (−3.36 to −3.48 mm and at −3.60 mm from the bregma). The CeA and Acb were also collected and assessed to verify injection sites. Two-channel fluorescent images of the BLA were obtained using an Axioscan 7 slide scanner (Carl Zeiss). For single-molecule FISH, three sections were analyzed from each animal: one middle (−2.92 to −3.12 mm from the bregma) and two posterior (−3.36 to −3.48 mm and at −3.60 mm from the bregma).

gCaMP7f and jRGECO1a expressions were verified using two-color fluorescence. The free-floating tissue was washed in phosphate buffer (PB) and nonspecific binding was blocked using a mixture of 2.5% normal donkey serum (NDS) and normal goat serum (NGS) in PB. The tissue was incubated in 1:1,500 chicken anti-GFP (Thermo Fisher Scientific, catalog #A10262, RRID:AB_2534023) and rabbit anti-RFP (Thermo Fisher Scientific, catalog #710530, RRID:AB_2532732) diluted in PBTX and 2% NGS and 2% NDS overnight in room temperature. Unbounded primary antibodies were washed off and then incubated in 1:1,000 Alexa Fluor 488 goat anti-chicken (Thermo Fisher Scientific, catalog #A-11039, RRID:AB_2534096) and 1:1,000 Alexa 594 donkey anti-rabbit (Thermo Fisher Scientific, catalog #A-21207, RRID:AB_141637) for 4 h in room temperature. Sections were washed and mounted onto frosted microscope slides and coverslipped with Permafluor (Thermo Fisher Scientific).

eGFP and eNpHR3.0 expressions were revealed using diaminobenzidine (DAB) immunochemistry. The free-floating tissue was washed and dehydrated in alcohol, and nonspecific binding was blocked (5% NHS in PB). Sections were incubated in 1:2,000 rabbit anti-GFP (Thermo Fisher Scientific) diluted in PBTX and 2% NHS for 24 h in room temperature. Sections were washed and incubated in 1:2,000 biotinylated donkey anti-rabbit (Jackson ImmunoResearch Laboratories) diluted in PBTX and 2% NHS overnight in room temperature. Sections were washed and incubated in avidin–biotin complex diluted in PBTX for 2 h in room temperature. Sections were washed and incubated in DAB solution for 15 min. Reactions were initiated by 0.2 µl/ml glucose oxidase aspergillus (Sigma-Aldrich) and stopped by washing sections with an acetate buffer. Sections were washed and mounted onto gelatin-coated microscope slides. Slides were dehydrated in ethanol and cleared in histolene and then coverslipped with Entellan (ProSciTech). For retrograde tracing verification, placements were confirmed using native fluorescence viewed under an Olympus BX52 fluorescent microscope.

### Statistics and data analyses

Fear was measured via freezing in the neutral context and via conditioned suppression of lever pressing in the reward context. Freezing (the absence of all movement except that required for breathing; Fanselow, 1994) was scored every 2 s, and the number of observations scored as freezing was converted to a percentage. Suppression ratios were calculated as CS lever presses / (pre-CS lever presses + CS lever presses), with pre-CS defined as the 1 min prior to the CS onset. A ratio of 0.5 indicates no suppression, as there are the same numbers of lever presses during the CS as well as the minute prior. A ratio of 0 indicates complete suppression of lever pressing during the CS. These data were analyzed via repeated measures ANOVA. For behavior in fiber photometry experiments, fear learning was assessed via single within-subject factor of trials. For behavior in optogenetic experiments, fear learning was assessed via between-subject factor of group (eNpHR vs eYFP) and within-subject factors of trials (or days) and a group × trials (or days) interaction.

Fiber photometry signals were extracted to MATLAB and downsampled (15.89 Hz). Each signal (isosbestic 405 nm, Ca^2+^-dependent 465 nm, and Ca^2+^-dependent 560 nm during dual-color photometry) was high-pass (90 s) and low-pass (1 Hz) filtered. Robust regression (Keevers et al., 2024) was used to fit the isosbestic signal onto Ca^2+^-dependent signals, and a separate df/*F* was calculated for gCaMP (green) and jRGECO1a (red) via (Ca2 + dependent signal − fitted 405 nm) / fitted 405 nm. df/*F* signals around the CS and US onset were isolated and aggregated; the 3 s before each event was used as a baseline, and the 7 s following each event was defined as the event transient.

Traditional approaches to analyze photometry data typically perform ANOVA on a summary statistic such as peak response (measured within some time window) and/or the integral of the response [i.e., the area under the curve (AUC)]. These approaches can be problematic. Researchers typically choose the analysis time window post hoc. If the window is too small, activity of interest is potentially missed; if the window is too large, the statistic loses meaningfulness. So, the analysis window is chosen after the experimenter has examined mean df/*F* around an event and/or examined the outcomes of analyses using different windows. This introduces unwanted biases into the analyses (“researcher degrees of freedom”), risking an inflated Type 1 error. Even when a suitable window is chosen, results only reveal whether overall activity within the window is significantly different to 0% df/*F*, not when in this window activity is significant, whether activity beyond this window is significantly different from 0% df/*F*, and the absence of a difference in peak or AUC data does not mean that periods of the transient are not statistically significant.

A bootstrapping confidence interval (bCI) procedure (95% CI, 1,000 bootstraps) overcomes these problems. We used a bCI procedure to determine significant event-related transients within this window (Jean-Richard-dit-Bressel et al., 2020). A distribution of bootstrapped df/*F* means was generated by randomly resampling from trial df/*F* waveforms, with replacement, for the same number of trials. A confidence interval was obtained per time point using the 2.5 and 97.5 percentiles of the bootstrap distribution, which was then expanded by a factor of sqrt(*n* / (*n* − 1)) to adjust for narrowness bias. Significant transients were defined as periods where 95% CI did not contain 0% df/*F* for at least 0.33 s (consecutive threshold).

The bCI procedure offers many advantages over traditional area under the curve or peak df/*F* analyses (Jean-Richard-dit-Bressel et al., 2020). First, the bCI procedure does not depend on post hoc decisions about which temporal window to use for an AUC analysis and uses the same window for all events. This reduces the possible influence of bias when selecting and reporting this window. Second, the bCI procedure provides additional information about the full timecourse of event-related transients showing when, during each event, the event-related transient is significant. Third, the bCI identifies the exact period of significant peak df/*F*. Fourth, by definition, the upper and lower bounds of the bCI provide interval estimates of the effect size. Fifth, the bCI has been demonstrated to control the Type 1 error rate at or below the nominated α and is statistically more powerful than other widely used analyses at the sample sizes typically used in photometry experiments (Jean-Richard-dit-Bressel et al., 2020). The cross-correlation analyses were conducted using MATLAB with df/*F* signals around the US onset (−3 to 7 s).

## Results

### BLA pathways are segregated

**Figure 1.**
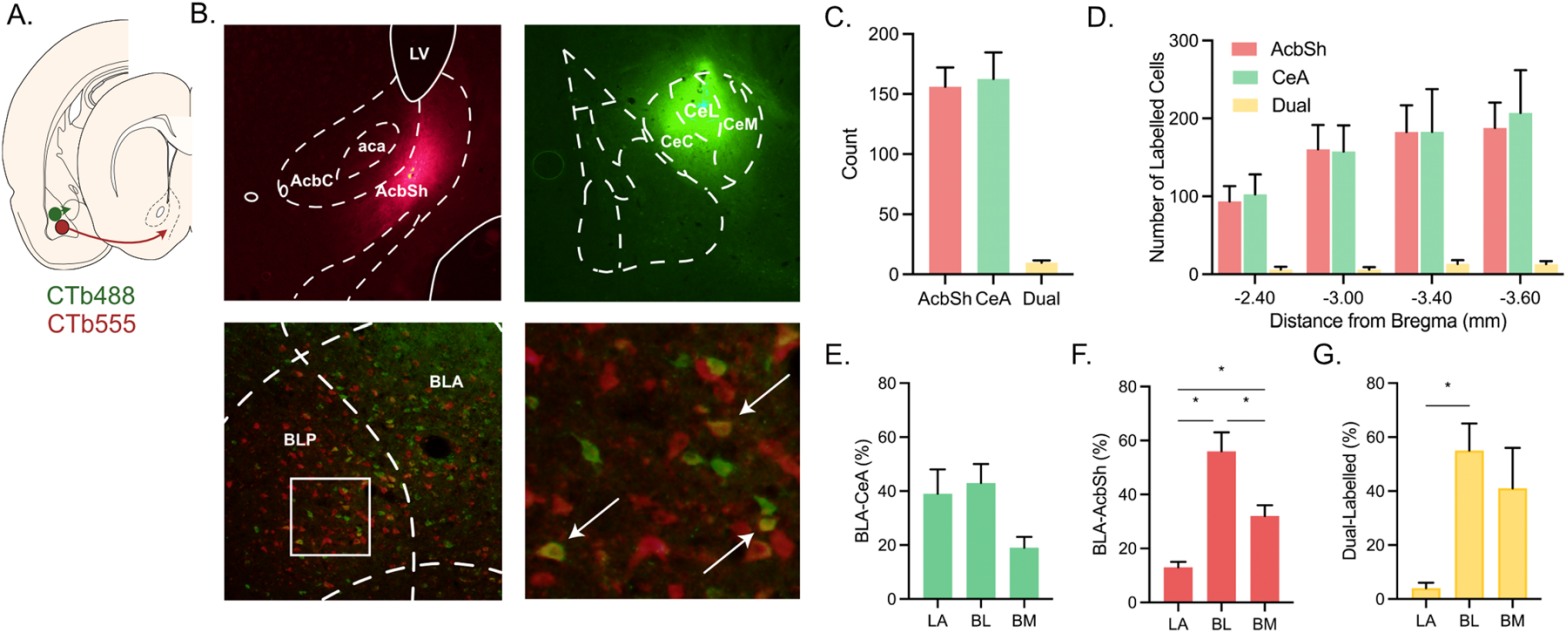
Segregation of BLA output pathways. ***A***, CTb-488 and CTb-555 were applied to CeA or Acb, and retrograde-labeled neurons in the BLA were identified. ***B***, Representative CTb deposits in the Acb, CeA, and retrograde-labeled neurons in the BLA. ***C***, Counts of single- and dual-labeled neurons in the BLA. ***D***, Anterior–posterior distribution of single- and dual-labeled neurons in the BLA. ***E–G***, Amygdala subnucleus distribution of single- and dual-labeled neurons.

First, we investigated the organization of BLA pathways to the Acb and CeA. We injected two differently colored retrograde tracers (*N* = 6), Alexa Fluor 488 and Alexa Fluor 555, into Acb and CeA and mapped retrograde-labeled neurons in the BLA (Fig. 1*A*,*B*). The vast majority (>97%) of labeled cells were single labeled, expressing only one of the two tracers, and there were significantly more single-labeled compared with double-labeled (∼3%) cells (*F*_(1,5)_ = 97.05; *p* < 0.001; Fig. 1*C*,*D*), confirming that these two pathways are mostly segregated in the rat, consistent with previous findings from mice (Beyeler et al., 2018). CeA- and Acb-projecting neurons were intermingled and distributed across the anterior–posterior axis of the BLA (Fig. 1*D*). They were also distributed across BLA subnuclei, but distinct subpopulations were preferentially located in different nuclei (Fig. 1*E*–*G*). CeA-projecting neurons were distributed across the rat BLA, with no differences across the lateral amygdala (LA), BLA, or basomedial amygdala (BM), largest (*F*_(1,5)_ = 4.30; *p* > 0.05). Meanwhile, there were more Acb-projecting neurons in the BL (*F*_(1,5)_ = 37.79; *p* = 0.002) and BM (*F*_(1,5)_ = 24.44; *p* = 0.004) than in the LA and more Acb-projecting neurons in the BL than the BM (*F*_(1,5)_ = 9.62; *p* = 0.027). For dual-labeled neurons, there was a greater percentage of neurons in the BL relative to the LA (*F*_(1,5)_ = 20.89; *p* = 0.006).

### BLA pathways share molecular profiles

**Figure 2.**
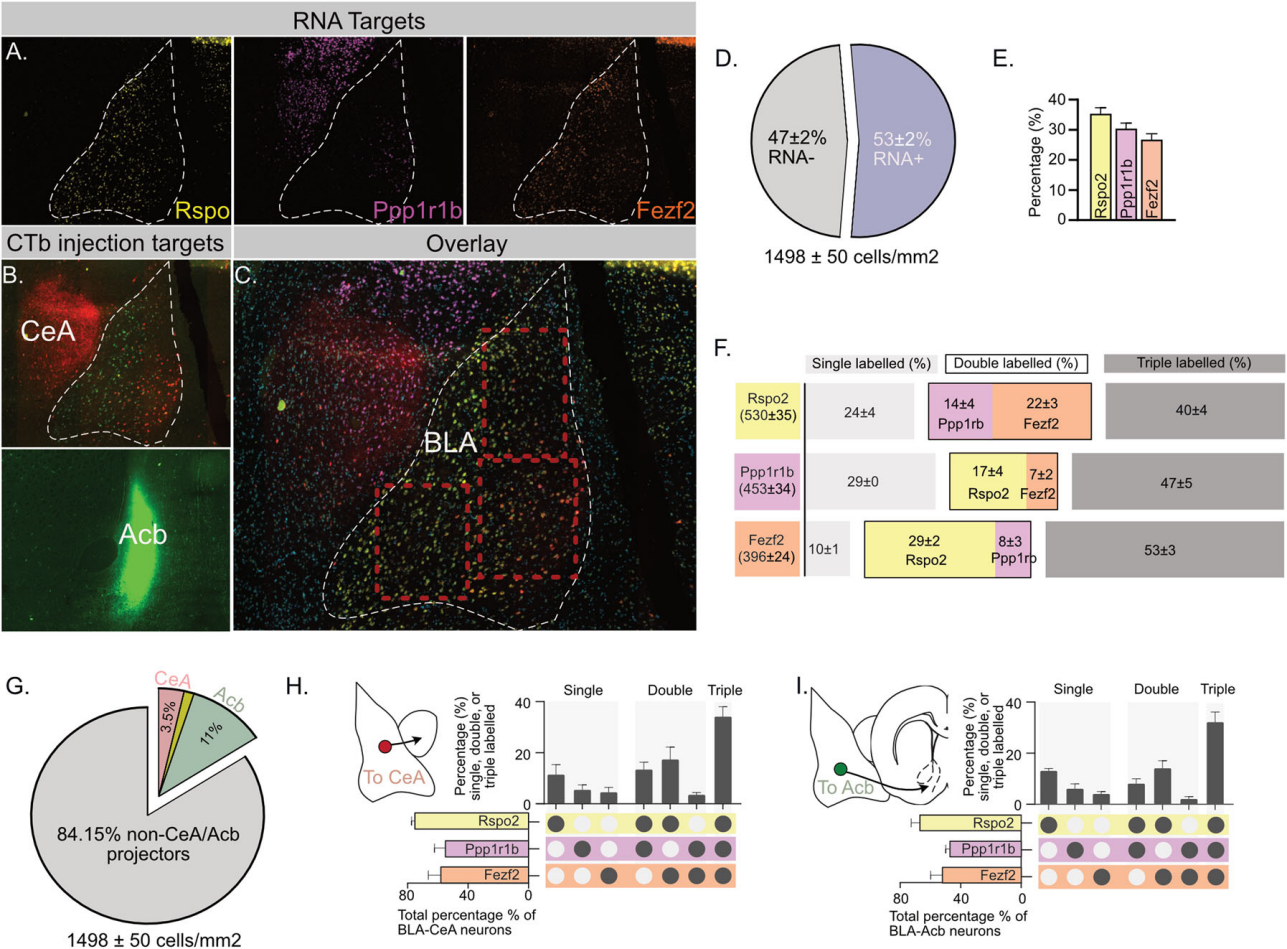
Molecular characterization of BLA output pathways. ***A***, Representative photomicrographs showing distribution of *Rspo*, *Ppp1r1b*, and *Fezf2* mRNA in rat BLA. ***B***, Representative CTb deposits in the Acb and CeA; ***C***, Overlay of retrograde-labeled neurons and *Rspo*, *Ppp1r1b*, and *Fezf2* mRNA in rat BLA. ***D***, The percentage of BLA neurons expressing any of *Rspo*, *Ppp1r1b*, and *Fezf2* mRNA. ***E***, The percentage of BLA neurons expressing either *Rspo*, *Ppp1r1b*, or *Fezf2* mRNA. ***F***, The percentage of BLA neurons single, double, or triple labeled for *Rspo*, *Ppp1r1b*, or *Fezf2* mRNA. ***G***, Percentages of BLA→CeA and BLA→Acb neurons. Single, double, or triple labeled for *Rspo*, *Ppp1r1b*, or *Fezf2* mRNA. Scale bars, 200 µm. ***H***, ***I***, UpSet plots showing combination of gene labeling in Acb-projecting (***H***) and CeA-projecting (***I***) BLA neurons.

Different functional BLA neuron populations have been distinguished based on the expression of distinct genetic markers in mice, most notably the genes *Rspo2*, *Ppp1r1b*, and *Fezf2* (Kim et al., 2016; Zhang et al., 2021). So, we asked whether these markers also distinguish between the BLA→CeA and BLA→Acb pathways in rats. We injected retrograde tracers into the rat (*N* = 4) CeA and Acb and used single-molecule FISH to label BLA for *Rspo2*, *Ppp1r1b*, and *Fezf2* mRNA expression (Fig. 2*A*–*C*). Approximately half of all BLA cells expressed at least one of these mRNA targets (Fig. 2*D*), confirming their abundant expression in the rat BLA. Interestingly, *Rspo2*, *Ppp1r1b*, and *Fezf2* were expressed at similar levels (*F*_(1,3)_ = 6.725; *p* > 0.05; Fig. 2*E*). Of note, most labeled cells were tripled labeled with all three gene targets (Fig. 2*F*). We then asked whether these gene expression profiles related to BLA→CeA and BLA→Acb pathways (Fig. 2*G*). We found the expression profiles of *Rspo2*, *Ppp1r1b*, and *Fezf2* were similar across the BLA→CeA and BLA→Acb pathways (Fig. 2*H*,*I*). A majority of neurons at the origin of each pathway expressed all three mRNAs (Kim et al., 2017; He et al., 2023; Madur et al., 2023). So, although *Rspo2*, *Ppp1r1b*, and *Fezf2* expression can distinguish valence coding neurons in the mouse BLA (Kim et al., 2016; Zhang et al., 2021), these three genes are highly colocalized in rat BLA and do not distinguish between the BLA→CeA and BLA→Acb pathways.

### BLA output pathways show circuit- and state-dependent US responsivity

The BLA has important roles in both positive and negative valence. These roles can be distinguished by different BLA circuits, with BLA→CeA pathway important for negative valence and BLA→Acb pathway important for positive valence (Namburi et al., 2015; Beyeler et al., 2016, 2018). This raises the possibility that these two pathways may act in opposition to each other during fear learning. However, whether these two BLA circuits have the same or different activity profiles during fear learning is unknown. Furthermore, whether activity profiles are determined by the conditions under which fear is learned is also unknown.

**Figure 3.**
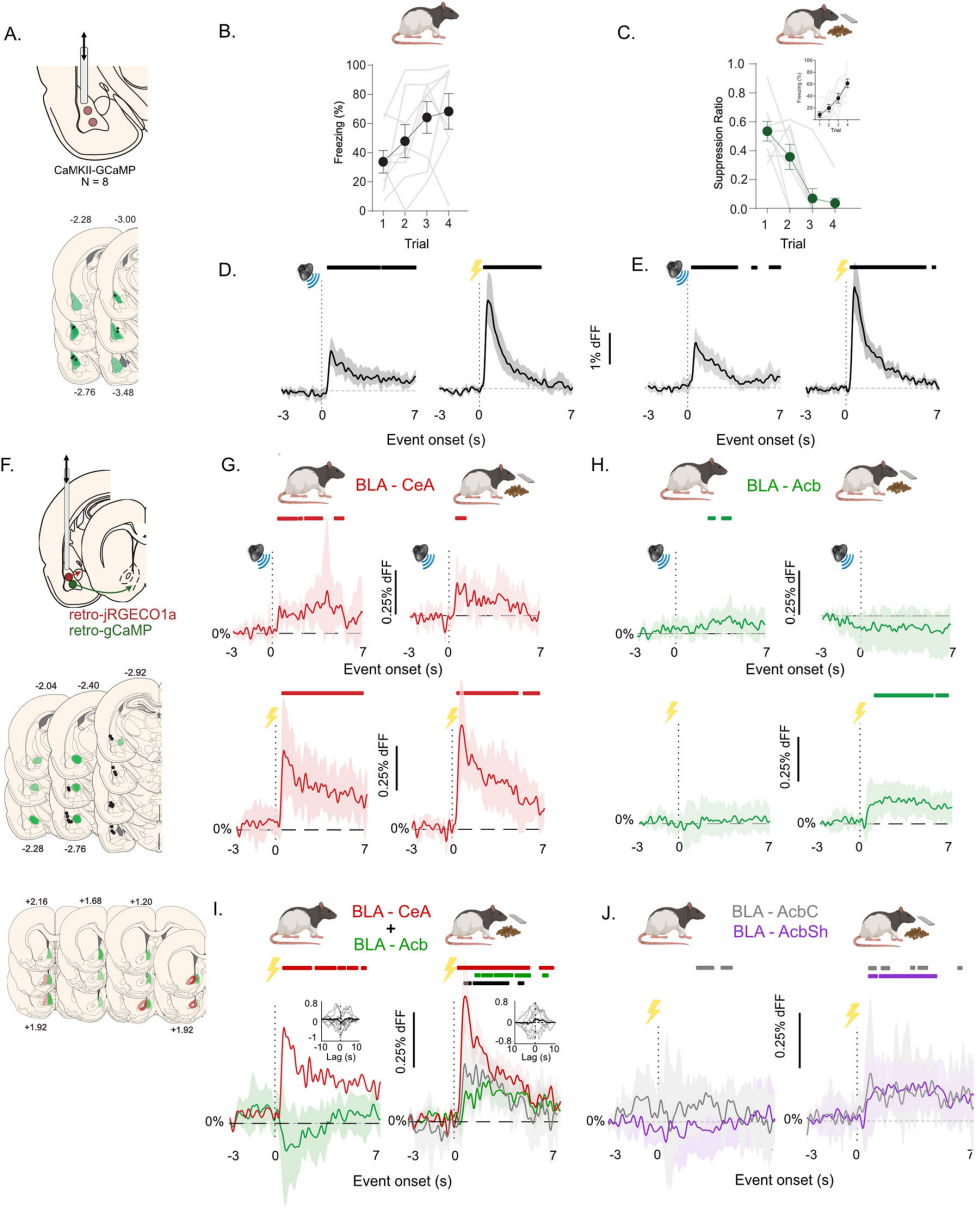
Activity profiles of BLA→CeA and BLA→Acb neurons during fear learning. ***A***, BLA application of CaMKIIa-gCaMP and location of AAV expression (green) as well as fiber tips (black dots) in BLA. ***B***, Acquisition of fear to the auditory CS in the neutral context. ***C***, Acquisition of fear to the auditory CS in the reward context as measured by conditioned suppression (main panel) or freezing (inset). ***D***, Mean and 95% bootstrapped confidence intervals for CS- and shock US-evoked transients during fear learning in the neutral context. ***E***, CS- and shock US-evoked transients during fear learning in the reward context. ***F***, Top row, CeA or Acb application of retro-jRGECO1a and retro-gCaMPCaMKIIa-gCaMP. Middle row, black dots show location of fibre tips; green shows location of CeA AAV injections. Bottom row, Location of AAV injections in Acb; green shows injections predominantly in AcbSh; red shows injections predominantly in AcbC. ***G***, Mean and 95% bootstrapped confidence intervals for CS- and shock US-evoked transients during fear learning in the neutral and reward context for BLA→CeA neurons. ***H***, Mean and 95% confidence intervals for CS- and shock US-evoked transients during fear learning in the neutral and reward context for BLA→Acb neurons. ***I***, Mean and 95% bootstrapped confidence intervals for shock US-evoked transients during fear learning in the neutral and reward context for dual-color BLA→CeA and BLA→Acb. The gray waveform is the within-subject mean difference (i.e., interaction) waveform for US-evoked transients in the BLA→Acb pathway in the two contexts with 95% bootstrapped confidence intervals. ***J***, Mean and 95% bootstrapped confidence intervals for shock US-evoked transients during fear learning in the neutral and reward context for BLA→AcbC and BLA→AcbSh neurons. Colored bars above waveforms indicate exact periods of statistically significant transients (%df/*F* > 0).

To answer these questions, we first injected CaMKIIα-gCaMP7f into the rat BLA (*N* = 8) and used fiber photometry to record from a global population of BLA neurons (Fig. 3*A*), while the same rats underwent two different forms of fear conditioning. In the neutral context, the conditioning chamber was bare except for the wall walls, roof, and grid floor, and rats received pairings of an auditory CS with a 0.5 mA, 0.5 s footshock US. Conditioned freezing was measured as the fear response to presentations of the auditory CS. In the reward context, a lever was available, and lever pressing led to a scheduled delivery of a food pellet to a magazine. Pairings of the auditory CS with a 0.5 mA, 0.5 s footshock US were superimposed on this lever pressing in a response-independent manner. Conditioned suppression of lever pressing was measured as the primary fear response, but we also measure freezing in this context. It is important to note that the contexts were matched on the total time rats had spent in them prior to and during conditioning. Rats acquired fear to both auditory CSs (Fig. 3*B*,*C*). In the neutral context, rats increased freezing to the auditory CS across training (main effect of trial, *F*_(1,7)_ = 12.222; *p* < 0.01). Likewise in the reward context, lever pressing rates (as shown by suppression ratios) decreased during the different auditory CSs across training showing the acquisition of conditioned suppression (main effect of trial, *F*_(1,7)_ = 53.226; *p* < 0.01), and freezing increased across trials (main effect of trial, *F*_(1,7)_ = 32.257; *p* < 0.001). Figure 3, *D* and *E* shows the mean ± 95% bootstrapped confidence intervals (i.e., full interval estimates) for CS and shock elicited transients in the neutral and reward contexts. To assess statistical significance of event-related Ca^2+^-transients, we used a 95% bCI procedure to estimate the periods when the 95% bCIs did not include 0% df/*F*, and so significantly differed from 0% df/*F* (see Materials and Methods). These periods are shown by colored bars above the Ca^2+^-transients in Figure 3. BLA neurons showed excitatory Ca^2+^-transients to the auditory CS and the footshock US in both neutral and reward contexts (Fig. 3*D,E*).

Next, we examined the activity of the BLA output pathways across the two different conditions of fear learning. We injected retrograde AAVs containing either gCaMP7f or jRGECO1a into the Acb and CeA and implanted a fiber optic above the BLA to record auditory CS-evoked and footshock US-evoked Ca^2+^-transients in a pathway-specific manner (Fig. 3*F*). BLA→CeA (*n* = 9) neurons showed robust excitatory Ca^2+^-transients to the auditory CS and footshock US in both the neutral and reward context (Fig. 3*G* shows mean ± 95% bootstrapped confidence intervals). In contrast, BLA→Acb (*n* = 20) neurons only showed excitatory Ca^2+^-transients to the footshock US in the reward context and showed no detectable Ca^2+^-transients to the footshock US in the neutral context (Fig. 3*H* shows mean ± 95% bootstrapped confidence intervals). BLA→Acb (*n* = 20) neurons also showed very modest excitatory transients to the auditory CS in the neutral context.

Importantly, we found a similar pattern of footshock US-evoked Ca^2+^-transients when examining the BLA→CeA and BLA→Acb pathways within the same subjects (*n* = 8). Taking advantage of the fact that our within-subject, dual-color photometry permits fair and direct comparison of footshock US Ca^2+^-transients between BLA→Acb pathway in the same animal in both contexts, we found that footshock US Ca^2+^-transients in the BLA→Acb pathway were significantly greater in the reward context compared with the neutral context (Fig. 3*I*; gray waveform is mean difference waveform ±95% bootstrapped confidence intervals) between the two pathways and the black bar above Figure 3*I* shows when this mean difference waveform does not include 0% df/*F*. It is important to note that this within-subject difference in shock US-evoked Ca^2+^-transients between the two contexts corresponds to a context (reward vs neutral) × time (0–7 s) interaction in footshock US Ca^2+^-transients for the BLA→Acb pathway. Cross-correlation analyses showed that Ca^2+^-transients were modestly anticorrelated in the neutral context but not in the appetitive context (Fig. 3*I*). Finally, we asked whether these profiles of footshock US-evoked Ca^2+^-transients varied across BLA neurons projecting to the core (AcbC) or shell (AcbSh) subregions of the Acb. We divided animals into two groups based on whether AAV injection site predominantly affected AcbC (*n* = 5) or AcbSh (*n* = 13; Fig. 3*J* shows mean ± 95% bootstrapped confidence intervals). Neither BLA→AcbC nor BLA→AcbSh neurons showed robust footshock US-evoked Ca^2+^-transients in the neutral context, but both showed robust footshock US-evoked Ca^2+^-transients in the reward context, with the BLA→AcbSh neurons in particular discriminating between the two contexts.

### BLA output pathways have opposing roles in fear learning

Our findings show that BLA neurons respond to a footshock in a circuit- and state-dependent manner. Footshock-evoked Ca^2+^-transients in the BLA→CeA pathway in both the neutral and reward contexts, whereas it only evoked transients in the BLA→Acb pathway in the reward context. Next, we used photoinhibition to determine the roles of this US-evoked activity in fear learning.

**Figure 4.**
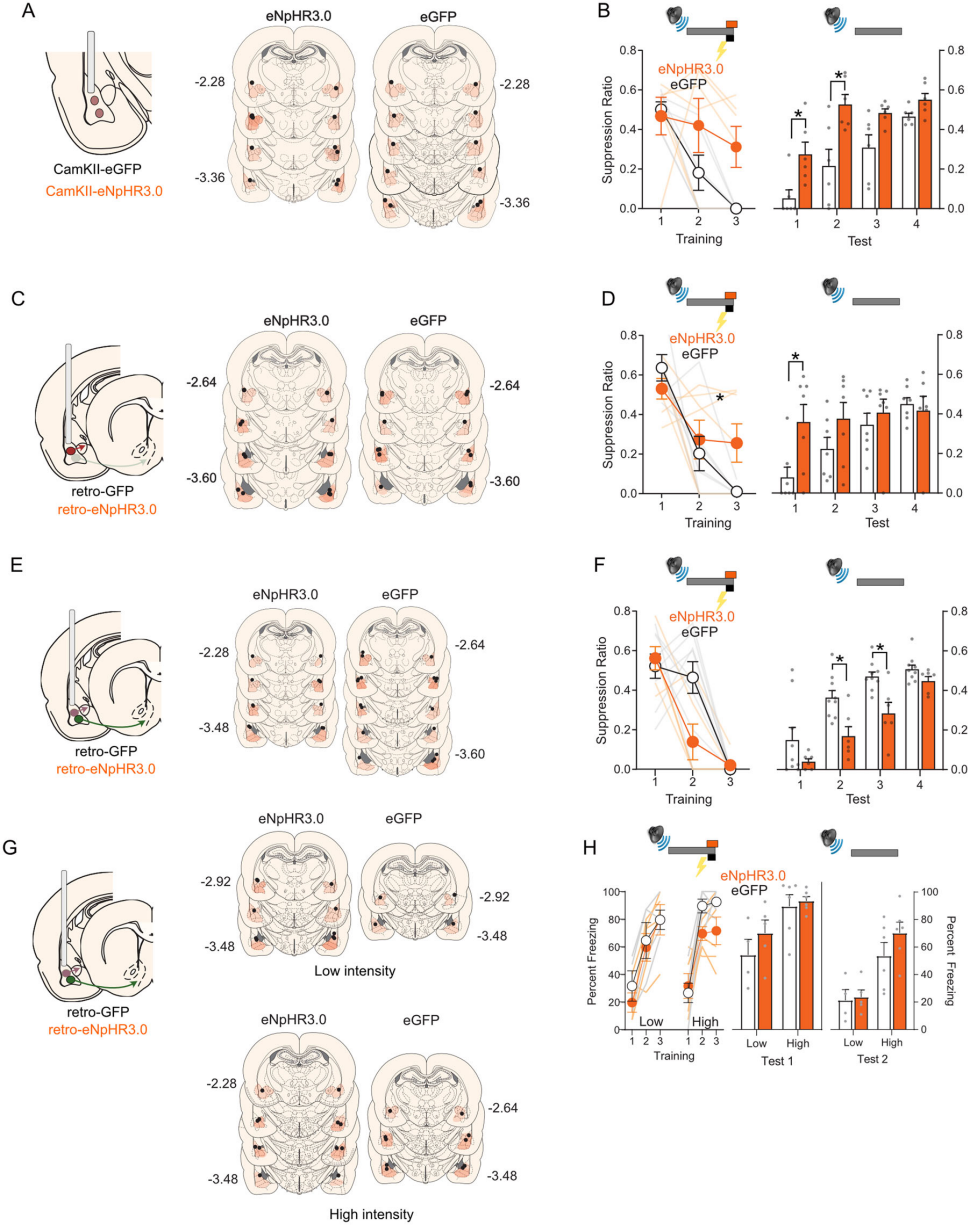
Photoinhibition of BLA output pathways and fear learning. ***A***, BLA application of CaMKIIa-eNpHR3.0 or CaMKIIa-eGFP and location of AAV expression (orange) as well as fiber tips (black dots) in BLA. ***B***, Photoinhibition of global BLA neurons during the shock US impaired fear learning in the reward context. ***C***, CeA application of retro-eNpHR3.0 or eGFP and location of AAV expression (orange) as well as fiber tips (black dots) in BLA. ***D***, Photoinhibition of BLA→CeA neurons during the shock US impaired fear learning in the reward context. ***E***, Acb application of retro-eNpHR3.0 or retro-eGFP and location of AAV expression (orange) as well as fiber tips (black dots) in BLA. ***F***, Photoinhibition of BLA→Acb neurons during the shock US augmented fear learning in the reward context. ***G***, Acb application of retro-eNpHR3.0 or retro-eGFP and location of AAV expression (orange) as well as fiber tips (black dots) in BLA. ***H***, Photoinhibition of BLA→Acb neurons during the shock US had no effect on fear learning in the neutral context. *p < 0.05.

First, we first sought to replicate past findings that footshock-evoked activity in a global population of BLA neurons is necessary for fear learning. Rats received CamKIIα-eNpHR3.0 (*n* = 6) or CamKIIα-eYFP (*n* = 6), fiber-optic implants into the BLA, and we photoinhibited BLA principal neurons at the time of the footshock during fear learning in the reward context (Fig. 4*A*,*B*). Rats acquired fear to the auditory CS, showing a significant increase in conditioned suppression across days of training (main effect of day, *F*_(1,10)_ = 17.57; *p* = 0.002). The overall difference in suppression between groups and the difference between groups across training approached but did not reach significance (main effect of group, *F*_(1,10)_ = 4.13; *p* = 0.069; group × day interaction, *F*_(1,10)_ = 4.847; *p* = 0.052). When subsequently tested for fear to the CS without photoinhibition, eNpHR3.0 rats showed significantly less fear compared with controls [main effect of group, *F*_(1,10)_ = 11.32; *p* = 0.007; including on the first (*F*_(1,10)_ = 8.345; *p* = 0.016) and second (*F*_(1,10)_ = 9.535; *p* = 0.011) test days], a loss of fear across days (day main effect, *F*_(1,10)_ = 107.46; *p* < 0.001), and no group × day interaction (*F*_(1,10)_ = 4.85; *p* > 0.05). This disruption of fear learning is consistent with previous work in mice (Wolff et al., 2014; Namburi et al., 2015) and rats (Johansen et al., 2014; Sengupta et al., 2016, 2018) and shows that the activity of BLA principal neurons at the time of the footshock US supports fear learning.

Next, we separately tested the roles of footshock-evoked activity in the BLA→CeA and BLA→Acb pathways in fear learning in the reward context. We asked how photoinhibition of BLA→CeA pathway at the time of the footshock affected fear learning. We injected the retrograde AAV containing eNpHR3.0 (*n* = 7) or the control eGFP (*n* = 7) into the bilateral CeA and implanted fiber-optic cannulae above the BLA (Fig. 4*C*) to silence BLA→CeA neurons at the time of the footshock US during fear learning (Fig. 4*D*). Rats acquired fear to the CS (main effect of day, *F*_(1,12)_ = 33.61; *p* < 0.001). There was no main effect of group (*F*_(1,12)_ = 1.31; *p* > 0.05), but fear learning was impaired by BLA→CeA photoinhibition because the eNpHR3.0 group showed slower rates of fear learning across days compared with controls (group × day interaction effect, *F*_(1,12)_ = 5.13; *p* = 0.04). Indeed, the eNpHR3.0 group showed significantly less fear to the CS compared with the eGFP group on the last day of fear acquisition (main effect of group, *F*_(1,12)_ = 6.33; *p* = 0.03). This impairment of learning was also evident when rats were tested for their fear to the CS without photoinhibition. There were no overall differences between groups on test (main effect of group, *F*_(1,12)_ = 1.80; *p *> 0.05), and both groups showed reductions in fear to the CS (main effect of trial, *F*_(1,12)_ = 21.06; *p *= 0.001). However, there was a group × trial interaction (*F*_(1,12)_ = 8.26; *p *= 0.01). Importantly, the eNpHR3.0 group showed significantly less fear to the CS on the first day of test compared with the eGFP group (main effect of group, *F*_(1,12)_ = 7.17; *p *= 0.02). So, footshock-evoked activity in the BLA→CeA pathway drives fear learning.

In a similar manner, we asked how photoinhibition of BLA→Acb pathway at the time of footshock affected fear learning (Fig. 4*E*,*F*). Rats acquired fear to the CS across learning (main effect of day, *F*_(1,13)_ = 121.19; *p* < 0.001). The overall difference in suppression between eNpHR3.0 (*n* = 6) and eGFP (*n* = 9) groups during training approached but did not reach significance (group main effect, *F*_(1,13)_ = 4.54; *p* = 0.052; group × day interaction, *F*_(1,13)_ = 0.04; *p* > 0.05). However, in contrast to the BLA→CeA pathway, BLA→Acb photoinhibition augmented fear learning because the eNpHR3.0 group showed significantly more fear to the CS across the test compared with controls (main effect group, *F*_(1,13)_ = 13.01; *p* = 0.003; Day 2 *F*_(1,13)_ = 10.23; *p* = 0.007; Day 3 *F*_(1,13)_ = 11.29; *p* = 0.005; main effect of day, *F*_(1,13)_ = 84.04; *p* < 0.001; group × day interaction, *F*_(1,13)_ = 0.19; *p* > 0.05). So, footshock-evoked activity in the BLA→Acb pathway opposes fear learning.

Our dual-color fiber photometry showed that footshock failed to evoke Ca^2+^-transients in the BLA→Acb pathway during fear learning in the neutral context, in contrast to the robust footshock-evoked Ca^2+^-transients observed in the reward context. This pathway-selective activation implies that silencing BLA→Acb neurons during footshock in the neutral context should have no effect on fear learning. To assess this, we injected the retrograde AAV containing eNpHR3.0 (*n* = 11) or the control eGFP (*n* = 10) into the bilateral Acb and implanted fiber-optic cannulae above the BLA (Fig. 4*G*) to silence BLA→Acb neurons at the time of the footshock during fear learning in a neutral context (Fig. 4*H*). We used two different footshock intensities during fear conditioning [0.35 mA (*n* = 9) or 0.7 mA (*n* = 12)] to ensure that any potential effect of fear learning was not masked by a floor or ceiling effect. Rats acquired fear to the CS across learning in both the low footshock intensity (main effect of day, *F*_(1,7)_ = 39.99; *p* < 0.001) and high footshock intensity (main effect of trial, *F*_(1,7)_ = 45.36; *p* < 0.001) conditions (Fig. 4*G*). However, there was no difference between eNpHR3.0 and eGFP groups in either condition (low US, main effect of group, *F*_(1,7)_ = 0.24; *p* > 0.05; high US, main effect of group, *F*_(1,10)_ = 3.28; *p* > 0.05; no group × trial interactions, all *F*s < 1; *p* > 0.05). There was also no difference between eNpHR3.0 and eGFP groups across test. On tests, the high US groups expressed more fear than the low US groups (main effect US, *F*_(1,17)_ = 26.15; *p* = 0.001), and fear decreased across tests (main effect day, *F*_(1,17)_ = 40.801; *p* < 0.001), confirming adequate variation in fear levels and statistical power to detect an augmentation of learning by photoinhibition, but fear learning did not differ between the eNpHR3.0 and eGFP groups (main effect of group, *F*_(1,17)_ = 2.05; *p* > 0.05; no group × US, group × day, or group × US × day interactions, all *F*s < 1.83; *p* > 0.05).

## Discussion

Here we showed that amygdala circuits are involved in state-dependent opponent processing of fear. First, we showed that BLA→CeA and BLA→Acb pathways are largely segregated in the BLA but share overlapping molecular profiles. Using fiber photometry, we showed, for the first time, that BLA→CeA neurons are robustly recruited by footshock regardless of where fear learning occurs, whereas the recruitment of BLA→Acb neurons is state-dependent because footshock only recruited this pathway in a reward context. Finally, using circuit-specific photoinhibition, we showed that this footshock recruitment of BLA→CeA and BLA→Acb circuits serves opposing functions in fear learning: activity of BLA→CeA neurons supports fear learning, whereas activity of BLA→Acb neurons opposes fear learning.

### State-dependent opponent processing of fear

Our key finding is that BLA aversive US processing is circuit- and state-dependent. The BLA→CeA pathway promotes fear learning. This pathway was recruited by the footshock US regardless of where fear conditioning occurred and silencing this pathway impaired fear learning regardless of where fear conditioning occurred. In contrast, the BLA→Acb pathway opposes fear learning. This pathway was recruited by the footshock US when fear was conditioned in a reward context, but not neutral context, and silencing this pathway augmented fear learning in the reward but not neutral context. This identification of the BLA→Acb pathway as a circuit substrate for a footshock US-elicited fear opponent process accords well with findings from rodents and humans implicating the Acb in the relief and safety occasioned by omission or termination of an aversive US (Leknes et al., 2011; Becerra et al., 2013; Mohammadi et al., 2014). It adds significantly to these findings by showing for the first time that the aversive footshock US can itself activate this fear opponent pathway.

Footshock could recruit the BLA→Acb pathway in the reward context but not neutral context. The precise driver(s) of this state-dependent recruitment will be important to determine. One possibility is that the reward context increased excitability of BLA→Acb neurons and hence the likelihood for these neurons to be recruited by the footshock. This is plausible because the BLA→Acb pathway has a critical role in reward behaviors (Stuber et al., 2011; Dieterich et al., 2021) and increased excitability of BLA neurons increases their likelihood to be recruited during learning (Yiu et al., 2014). This would be consistent with other evidence that excitability of BLA→CeA and BLA→Acb neurons is modulated by recent experiences, including learning and changes in internal states. For example, fear conditioning strengthens the BLA→CeA pathway but weakens the BLA→Acb pathway, whereas reward learning has the opposite effect (Namburi et al., 2015). Food deprivation increased baseline activity of BLA→Acb neurons but decreased baseline activity of BLA→CeA neurons. Furthermore, food deprivation switched the relationship between BLA→CeA and BLA→Acb neurons, with BLA→CeA photostimulation inhibiting BLA→Acb neurons in nondeprived mice but exciting BLA→Acb neurons in food-deprived mice (Calhoon et al., 2018). It is worth emphasizing that food restriction per se is not the critical variable dictating recruitment of the BLA→Acb pathway by footshock here. We used a within-subject design to control for this possibility, with concurrent measurement of the BLA→CeA and BLA→Acb pathways in the same animals under the same food restriction regimes. These findings show that interactions between the BLA→CeA and BLA→Acb pathways are not invariant and are consistent with our finding of circuit- and state-dependent opponent processing of fear.

Showing functional interactions between BLA→CeA and BLA→Acb pathways, our results do not speak to precise loci of these interactions. One possibility is that there are direct opponent interactions between the distinct populations of BLA principal neurons projecting to the CeA and Acb, likely via BLA interneurons (Polepalli et al., 2010; Wolff et al., 2014; Yau et al., 2021). Such interactions have been shown in vitro (Kim et al., 2016) and in vivo (Piantadosi et al., 2024) for BLA valence coding neurons. Whether these interactions extend to projection-defined BLA neuron populations will be an important question for future work. Equally interesting will be when the fear opponent role of the BLA→Acb pathway is unmasked, for example, whether excitation of the BLA→Acb pathway, in the absence of prior reward training, can also limit how much fear is learned. This understanding could prove useful in designing novel interventions to minimize the fear learned after a traumatic experience.

Interestingly, none of the molecular markers we used (*Rspo2*, *Ppp1r1b*, and *Fezf2*) were able to distinguish fear opponent BLA→Acb neurons from fear promoting BLA→CeA neurons. This was surprising because these markers have proved useful in delineating valence coding neurons in the mouse BLA but were coexpressed here in projection-defined rat BLA→Acb and BLA→CeA neurons. This suggests that there is likely is a complex and nuanced relationship between BLA neuron molecular identity, valence coding, and output pathway organization. For example, it is possible that the US-elicited fear opponent process identified here is distinct from, or due to only a subset of, BLA valence coding neurons. One marker that could be worth examination is *Thy-1*. Excitation of *Thy-1*–expressing BLA neurons at the time of the footshock US inhibits fear learning (Jasnow et al., 2013)—a functional profile consistent with a fear opponent process—and BLA *Thy-1* neurons also project to Acb (Porrero et al., 2010). So, BLA *Thy-1* neurons may be a cellular substrate of the fear opponent process discovered here.

### Methodological considerations

There are two issues to consider. First, we used dual-color photometry via jRGECO and GCaMP to assess footshock US-evoked activity in BLA→CeA and BLA→Acb neurons. A key consideration is spectral separation of these signals. It is unlikely that a lack of spectral separation can explain our findings: we counterbalanced which calcium indicator was expressed in the two pathways, we recorded during the same footshock events in the same animals in the reward and neutral context, and the state- and circuit-dependent effects of photoinhibition supported findings of state and circuit-dependent recruitment. Second, our experiments were restricted to male animals due to unavailability of female animals. It is important to note that many of our findings (global BLA footshock-evoked transients, effects of global BLA silencing, effects of BLA→CeA silencing) recapitulated past work in both sexes in rats and mice, so there are strong grounds for assuming generality of our findings. However, given that there are sex differences in activity of amygdala neurons linked to differences in excitatory synaptic input (Blume et al., 2017), this will remain an important empirical question.

## Conclusions

Here we show that BLA aversive US processing is circuit- and state-dependent. Whereas the BLA→CeA pathway was recruited by the aversive footshock US regardless of where fear learning occurred, the BLA→Acb pathway was recruited by the footshock US only in the reward context. This footshock-driven activity in the BLA→Acb pathway limits how much fear is learned.
